# Enhanced Prefrontal Neuronal Activity and Social Dominance Behavior in Postnatal Forebrain Excitatory Neuron-Specific *Cyfip2* Knock-Out Mice

**DOI:** 10.3389/fnmol.2020.574947

**Published:** 2020-10-29

**Authors:** Yinhua Zhang, Rim Kang Hyae, Seung-Hyun Lee, Yoonhee Kim, Ruiying Ma, Chunmei Jin, Ji-Eun Lim, Seoyeon Kim, Yeju Kang, Hyojin Kang, Su Yeon Kim, Seok-Kyu Kwon, Se-Young Choi, Kihoon Han

**Affiliations:** ^1^Department of Neuroscience, College of Medicine, Korea University, Seoul, South Korea; ^2^Department of Biomedical Sciences, College of Medicine, Korea University, Seoul, South Korea; ^3^Department of Physiology, Dental Research Institute, Seoul National University School of Dentistry, Seoul, South Korea; ^4^Division of National Supercomputing, Korea Institute of Science and Technology Information, Daejeon, South Korea; ^5^Center for Functional Connectomics, Korea Institute of Science and Technology, Brain Science Institute, Seoul, South Korea

**Keywords:** CYFIP2, medial prefrontal cortex, neuronal activity, social dominance, excitability

## Abstract

The cytoplasmic fragile X mental retardation 1 (FMR1)-interacting protein 2 (*CYFIP2*) gene is associated with epilepsy, intellectual disability (ID), and developmental delay, suggesting its critical role in proper neuronal development and function. CYFIP2 is involved in regulating cellular actin dynamics and also interacts with RNA-binding proteins. However, the adult brain function of CYFIP2 remains unclear because investigations thus far are limited to *Cyfip2* heterozygous (*Cyfip2^+/−^*) mice owing to the perinatal lethality of *Cyfip2*-null mice. Therefore, we generated *Cyfip2* conditional knock-out (cKO) mice with reduced CYFIP2 expression in postnatal forebrain excitatory neurons (*CaMKIIα-Cre*). We found that in the medial prefrontal cortex (mPFC) of adult *Cyfip2* cKO mice, CYFIP2 expression was decreased in both layer 2/3 (L2/3) and layer 5 (L5) neurons, unlike the L5-specific CYFIP2 reduction observed in adult *Cyfip2^+/−^* mice. Nevertheless, filamentous actin (F-actin) levels were increased only in L5 of *Cyfip2* cKO mPFC possibly because of a compensatory increase in CYFIP1, the other member of CYFIP family, in L2/3 neurons. Abnormal dendritic spines on basal, but not on apical, dendrites were consistently observed in L5 neurons of *Cyfip2* cKO mPFC. Meanwhile, neuronal excitability and activity were enhanced in both L2/3 and L5 neurons of *Cyfip2* cKO mPFC, suggesting that CYFIP2 functions of regulating F-actin and excitability/activity may be mediated through independent mechanisms. Unexpectedly, adult *Cyfip2* cKO mice did not display locomotor hyperactivity or reduced anxiety observed in *Cyfip2^+/−^* mice. Instead, both exhibited enhanced social dominance accessed by the tube test. Together, these results provide additional insights into the functions of CYFIP2 in the adult brain.

## Introduction

Two members of the cytoplasmic fragile X mental retardation 1 (FMR1)-interacting protein family, CYFIP1 and CYFIP2 (also referred to as SRA1 and PIR121, respectively), are evolutionarily highly conserved proteins which were originally identified as direct binding partners of FMR Protein (Schenck et al., 2001), an RNA-binding protein whose loss causes fragile X syndrome (Bagni and Zukin, 2019). Moreover, CYFIP1 and CYFIP2 are critical components of the Wiskott–Aldrich syndrome protein family verprolin-homologous protein (WAVE) regulatory complex (WRC; Chen et al., 2010), a heteropentameric protein complex consisting of CYFIP (either CYFIP1 or CYFIP2), WAVE, NAP1, ABI, and HSPC300. As a downstream effector of Rac1 GTPase, the WRC regulates Arp2/3 complex-mediated actin polymerization in various cellular compartments including neuronal synapses (Spence and Soderling, 2015; Chen et al., 2017).

Based on the above-mentioned protein interactions, CYFIP1 and CYFIP2 are known to be involved in the regulation of RNA-processing and actin dynamics, which are two critical molecular pathways for proper neuronal development and function (Bagni and Zukin, 2019; Zhang et al., 2019b). Notably, however, detailed *in vivo* studies of CYFIP1 and CYFIP2 have suggested their functions differ because they have distinct spatiotemporal expression patterns and different protein interactors in the brain (Zhang et al., 2019a; Lee Y. et al., 2020; Lee S. H. et al., 2020). Indeed, at least for survival, CYFIP1 and CYFIP2 cannot compensate for each other considering *Cyfip1*-null mice (Chung et al., 2015) and *Cyfip2*-null mice (Kumar et al., 2013; Han et al., 2015; Zhang et al., 2018) are nonviable owing to lethality at different developmental time points (early embryonic and perinatal lethality, respectively).

Both *CYFIP1* and *CYFIP2* genes are associated with various brain disorders (Abekhoukh and Bardoni, 2014; Zhang et al., 2019b). For example, deletions and duplications of *CYFIP1* are associated with autism spectrum disorders, intellectual disability (ID), and schizophrenia (Yoon et al., 2014; Oguro-Ando et al., 2015). Large chromosomal deletions harboring *CYFIP2* have been identified in patients with developmental delay, ID, and seizures (Spranger et al., 2000; Lee et al., 2016). Moreover, recent whole-exome and whole-genome sequencing studies have identified *de novo* *CYFIP2* variants in patients with developmental delay, ID, and early-onset epileptic encephalopathy (Nakashima et al., 2018; Peng et al., 2018; Lee et al., 2019b; Zhong et al., 2019; Zweier et al., 2019).

To understand the neurobiological mechanisms underlying *CYFIP*-associated brain disorders, several rodent models have been generated and characterized for both CYFIP1 (Bozdagi et al., 2012; Pathania et al., 2014; Chung et al., 2015; Oguro-Ando et al., 2015; Hsiao et al., 2016; Bachmann et al., 2019; Davenport et al., 2019; Domínguez-Iturza et al., 2019; Silva et al., 2019) and CYFIP2 (Han et al., 2015; Lee S. H. et al., 2020). Nevertheless, in most of these studies, investigations on the adult brain functions of CYFIP1 and CYFIP2 were limited to the heterozygous mice (*Cyfip1^+/−^* or *Cyfip2^+/−^*) because of early developmental lethality of *Cyfip1*- and *Cyfip2*-null mice. In the case of CYFIP1, a conditional knock-out (cKO) strategy combining floxed-*Cyfip1* mice and brain region-specific Cre-expressing mice was applied to reveal inhibitory synaptic functions of the protein (Davenport et al., 2019).

We recently reported prefrontal dysfunctions in adult *Cyfip2^+/−^* mice, including increased filamentous actin (F-actin), enlarged dendritic spines, and enhanced excitatory synaptic transmission and excitability (Lee S. H. et al., 2020). Notably, these changes were restricted to layer 5 (L5), but not in layer 2/3 (L2/3), neurons of the medial prefrontal cortex (mPFC) possibly because of a reduction in CYFIP2 protein exclusively in these L5 neurons of adult *Cyfip2^+/−^* mPFC. Importantly, it is not uncommon for protein levels to be unaffected when one copy of the gene is deficient. To overcome this, in this study, we crossed floxed-*Cyfip2* mice with *CaMKIIα-Cre* mice to generate *Cyfip2* cKO mice with reduced CYFIP2 expression specifically in postnatal forebrain excitatory neurons. *Cyfip2* cKO mice displayed many of the common phenotypes that are observed in *Cyfip2^+/−^* mice, but with some distinct molecular, cellular, and behavioral abnormalities, providing additional insights into understanding the *in vivo* brain functions of CYFIP2.

## Materials and Methods

### Mice

The floxed-*Cyfip2* and *Cyfip2^+/−^* mice used in this study have been previously described (Han et al., 2015; Zhang et al., 2018, 2019a; Lee S. H. et al., 2020). The *CaMKIIα-Cre* [B6.Cg-Tg(Camk2a-cre)T29-1Stl/J] and *Thy1-YFP* [B6.Cg-Tg(Thy1-YFP)HJrs/J] mice were obtained from the Jackson Laboratory. The mice were bred and maintained on a C57BL/6J background according to the Korea University College of Medicine Research Requirements. All procedures were approved by the Committee on Animal Research at Korea University College of Medicine (KOREA-2018-0174). The mice were fed *ad libitum* and housed under a 12 h light-dark cycle. All experiments were performed with an adult (8 to 10-week old) male *Cyfip2* mice and their littermate controls.

### RNA Purification and qRT-PCR

Real-time quantitative reverse transcription PCR (qRT-PCR) was performed as described previously (Choi et al., 2015; Jin et al., 2018; Lee et al., 2019a). Briefly, total RNA was extracted from the brain tissues using a miRNeasy Minikit (Qiagen) and two micrograms of total RNA were used for cDNA synthesis using the iScript™ cDNA Synthesis Kit (Bio-Rad). Target mRNAs were detected and quantified by a real-time PCR instrument (CFX96 Touch, Bio-Rad) using the SYBR Green master mix (Bio-Rad). The primer sequences for real-time PCR were described previously (Lee S. H. et al., 2020).

### Biochemistry and Antibodies for Western Blotting

Whole and synaptosomal lysates of the mouse brains were prepared as described previously (Han et al., 2009; Jin et al., 2019a,b). The antibodies used for Western blot analysis were anti-Cofilin (1:1,000, Abcam, AB42824), anti-CYFIP1 (1:1,000, Millipore, AB6046), anti-CYFIP2 (1:3,000, Abcam, AB95969), anti-GABA-A-R-β2/3 (1:1,000, NeuroMab, 75-363), anti-GAPDH (1:3,000, Cell Signaling, #2118), anti-Gephyrin (1:500, Synaptic Systems, 147-011), anti-GluA1 (1:2,000, Millipore, 04-855), anti-GluA2 (1:1,500, Millipore, MAB397), anti-GluN1 (1:1,000, Millipore, MAB363), anti-Homer1b/c (1:1,000, Synaptic Systems, 160-002), anti-Neuroligin-3 (1:1,000, NeuroMab, 75-158), anti-PSD-95 (1:2,000, Thermo Fisher Scientific, MA1-046), and anti-WAVE1 (1:1,000, NeuroMab, 75-048). Western blot images were acquired using a ChemiDoc Touch Imaging System (Bio-Rad) and quantified using ImageJ software.

### Immunohistochemistry

Mice were deeply anesthetized with isoflurane and transcardially perfused with heparinized (20 units/ml) phosphate-buffered saline (PBS) followed by 4% paraformaldehyde (PFA) in PBS. Brains were extracted and post-fixed in 4% PFA overnight. After post-fixation, the brains were washed with PBS and cryoprotected with 30% sucrose in PBS for 48 h. Brains were frozen in O.C.T compound (Sakura Tissue-Tek, 4583) and coronally sectioned (60 μm thickness) using a cryostat microtome (Leica, CM3050S). The sections were collected and stored in 50% glycerol in 2× PBS at −20°C until further processed. After being slightly rinsed in 1× PBS, sections were permeabilized by 0.5% Triton X-100 in 1× PBS three times for 10 min each. The sections were incubated with blocking solution (3% BSA in 0.5% Triton X-100 in 1× PBS) for 30 min at room temperature. Then, the sections were incubated with primary antibodies diluted in blocking solution overnight at 4°C. After washed three times for 15 min each with washing buffer (0.1% Triton X-100 in 1× PBS), the sections were incubated with Alexa Fluor-conjugated secondary antibodies diluted in blocking solution for 1 h at room temperature. The sections were washed with washing buffer three times for 15 min each. Finally, the sections were mounted on slide glasses with mounting media containing DAPI (Vector Laboratories, H-1400). The slide glasses were stored at 4°C until imaged. The list of primary and secondary antibodies used for immunohistochemistry is summarized in Supplementary Table 1. Confocal microscopy (Zeiss, LSM800) was used to acquire images (×20 objective and ×1 digital zoom) of the prelimbic mPFC (3.00–1.77 mm anterior from the Bregma) from coronal sections (Supplementary Figure 1). The prelimbic mPFC region was obtained by tile scanning (total of three tiles), and each frame was taken in *Z*-stacks of 5–10 slices (45–55 μm thickness in total). Using the anti-NeuN antibody, we examined antibody penetration to the mPFC section (Supplementary Figure 2). Tiled *Z-projection* images were aligned and converted into a single flattened image using ZEN software from Zeiss. From each tiled image, three to four regions of interest (ROI, 150 × 150 μm) were randomly selected from each cortical layer and were analyzed using ImageJ software. Specifically, CYFIP1 and cFos intensities in the cell body area of each ROI were measured and normalized by NeuN intensity for each cell body (Supplementary Figure 3A). For CYFIP2 and F-actin, the total intensity of each ROI was measured and normalized by total NeuN intensity for each ROI (Supplementary Figure 3B). All quantifications were performed by researchers who were blinded to the genotype and were involved in neither staining nor image acquisition processes.

### Dendritic Spine Analysis

Mice were deeply anesthetized with isoflurane and transcardially perfused with heparinized (20 units/ml) PBS followed by 4% PFA in PBS. Brains were extracted and post-fixed in 4% PFA overnight. After post-fixation, coronal sections (100 μm thickness) of the mPFC region (3.00–1.77 mm anterior to Bregma) were obtained using a vibratome (VT1000S, Leica). The sections were collected and stored in 50% glycerol in 2 × PBS at −20°C until further processed. Blocking, permeabilization, and anti-GFP (1:1,000, Abcam, AB13970) primary and Alexa Fluor-conjugated (1:500, anti-chicken Alexa Fluor-488) secondary antibody incubation were performed as described above. Finally, the sections were mounted on slide glasses with mounting media (Biomeda, M02). Images of dendritic spines on the secondary or tertiary branches (apical or basal dendrites of YFP-positive L5 neurons in the prelimbic mPFC) were acquired by confocal microscopy (Zeiss, LSM800, ×63 objective, and ×3 digital zoom) and were analyzed using ImageJ software. All quantifications were performed by researchers who were blinded to the genotype and were involved in neither staining nor image acquisition processes.

### Electrophysiology

Coronal slices of the mPFC were prepared (300 μm thickness) as described previously (Seo et al., 2012; Kang et al., 2017). After decapitation, brains were rapidly removed and placed in an ice-cold, oxygenated (95% O_2_ and 5% CO_2_), low-Ca^2+^/high-Mg^2+^ dissection buffer containing (in mM) 5 KCl, 1.23 NaH_2_PO_4_, 26 NaHCO_3_, 10 dextrose, 0.5 CaCl_2_, 10 MgCl_2_, and 212.7 sucrose. Slices were transferred to a storage chamber in an incubator containing oxygenated artificial cerebrospinal fluid (ACSF) containing (in mM) 124 NaCl, 2.5 KCl, 1.23 NaH_2_PO_4_, 2.5 CaCl_2_, 1.5 MgCl_2_, 26 NaHCO_3_, and 10-dextrose at 28–30°C for at least 30 min before recording. Slices were transferred to a recording chamber where they were perfused continuously with oxygenated ACSF (23–25°C) at a flow rate of 2 ml/min. Slices were equilibrated for 5 min before recordings. All recordings were performed in visually identified L2/3 or L5 pyramidal neurons in the prelimbic mPFC which were identified by their size and morphology. Patch pipettes (4–6 MΩ) were filled with (in mM) 145 K-gluconate, 5 NaCl, 10 HEPES, 0.2 EGTA, 1 MgCl_2_, 2 ATP-Mg, and 0.1 GTP-Na at a pH 7.4 and 280–290 mOsm. The extracellular recording solution consisted of ACSF supplemented with picrotoxin (100 μM). Data were acquired using an EPC-8 amplifier (HEKA), filtered at 3 kHz, digitized at 10 kHz with Digidata 1550B (Axon Instruments), and analyzed using pClamp 10 (Molecular Devices). Only cells with access resistance <20 MΩ and input resistance >100 MΩ were studied. Cells were discarded if the input or access resistance changed by more than 20%.

### Behavioral Assays

Before each test, mice were habituated in the test room (400 lux, 60 dB white noise) for at least 30 min. The open-field test was performed as described previously (Han et al., 2013, 2015). Briefly, mice were placed in the center of a clear, open Plexiglas chamber (40 × 40 × 30 cm), and their activities were recorded for 30 min using a suspended digital camera and analyzed using the EthoVision video tracking software (Noldus). The center area was defined as a 20 × 20 cm zone in the middle of the chamber. The elevated plus-maze test was performed using a plus-maze raised 50 cm above the floor. Mice were placed at the intersection of the four arms and allowed to move freely for 10 min. The tube test was performed as described previously (Fan et al., 2019) using a transparent acryl tube with 30 cm length and 3 cm inside diameter, a size just sufficient to permit only one adult mouse to pass through without reversing direction. For the training session, each mouse was released at alternating ends of the tube and allowed to run through the tube. Each animal was given 10 training trials on each of two successive days. For the test session, a pair of mice were released at the two ends of a tube and allowed to stop at a guillotine door in the middle of the tube. During the test session (2 min maximum), the guillotine door was opened and the mouse that retreated out of the tube first was designated as the loser.

### Gene Ontology and Pathway Analysis

Enrichment analysis was performed using the Enrichr web tool (Kuleshov et al., 2016) on Gene Ontology (GO) and Kyoto Encyclopedia of Genes and Genomes (KEGG) gene set libraries. Enrichr employs pre-defined gene set libraries to assist functional enrichment analysis of large gene lists. The enrichment of biological terms associated with the query list was assessed by Fischer’s exact test and the inferred *P*-values were further adjusted with Benjamini–Hochberg for multiple hypotheses testing. The enrichment terms with adjusted *P*-value less than 0.05 were considered significantly enriched.

### Quantification and Statistical Analysis

Values from at least four independent experiments with biological replicates were used for quantification and statistical analysis. All quantifications were performed in a blinded manner. *P* values were calculated by two-tailed student’s *t*-test or analysis of variance (ANOVA) with Bonferroni’s *post hoc* test using the GraphPad Prism 6 software. All data are presented as mean ± SEM. *P < 0.05; **P < 0.01; ***P < 0.001.

## Results and Discussion

**Figure 1:**
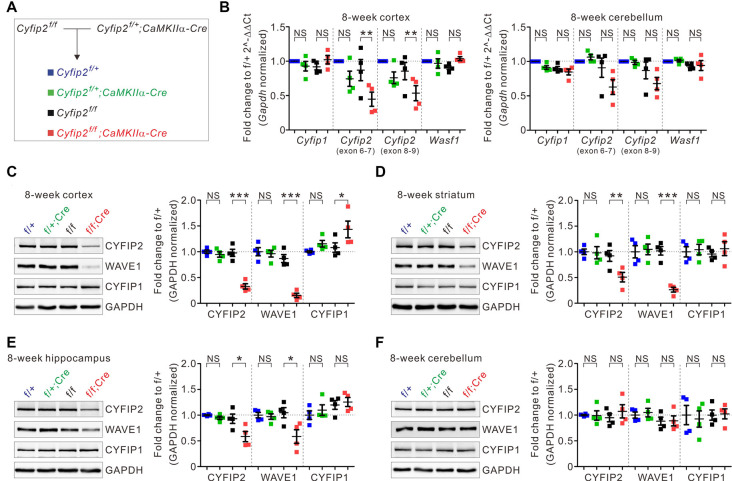
Changes in the expression of cytoplasmic fragile X mental retardation 1 (FMR1)-interacting protein 1 (*CYFIP1*), CYFIP2, and Wiskott–Aldrich syndrome protein family verprolin-homologous protein 1 (WAVE1) in multiple brain regions of *Cyfip2* conditional knock-out (cKO) mice. **(A)** The breeding scheme for the *Cyfip2* cKO mice. **(B)** Real-time quantitative reverse transcription PCR (qRT-PCR) analyses of *Cyfip1*, *Cyfip2*, and *Wasf1* (encoding WAVE1) mRNAs in the cortex (left panel) and cerebellum (right panel) of *Cyfip2* cKO mice (*n* = 4 animals per genotype, one-way analysis of variance (ANOVA) with Bonferroni’s *post hoc* test). NS, not significant. **(C–F)** Western blot analysis of CYFIP1, CYFIP2, and WAVE1 proteins in the cortex, striatum, hippocampus, and cerebellum of *Cyfip2* cKO mice (*n* = 4 animals per genotype, one-way ANOVA with Bonferroni’s *post hoc* test). All data are presented as mean ± SEM. *P < 0.05; **P < 0.01; ***P < 0.001.

To generate postnatal forebrain excitatory neuron-specific *Cyfip2* cKO mice, we crossed floxed-*Cyfip2* (*Cyfip2*^f/f^) mice (Lee S. H. et al., 2020) with *CaMKIIα-Cre* mice, which start expressing Cre recombinase in the forebrain excitatory neurons during the third to fourth postnatal week (Kim et al., 2018; Figure 1A). As expected from the Cre expression pattern, qRT-PCR analyses showed reduced mRNA expression of *Cyfip2* in the cortex, but not in the cerebellum, of adult (postnatal 8 weeks) *Cyfip2* cKO (*Cyfip2^f/f^*;CaMKIIα-Cre) mice compared with those of Cre-negative control (*Cyfip2*^f/f^) mice (Figure 1B). However, cortical mRNA expression of *Cyfip2* was not significantly decreased in *Cyfip2* conditional heterozygous (*Cyfip2*^f/+^;*CaMKIIα-Cre*) mice compared with those of respective Cre-negative (*Cyfip2*^f/+^) control mice. Moreover, mRNA expression levels of *Cyfip1* and *Wasf1* (encoding WAVE1) were not changed in the cortex of *Cyfip2* cKO mice. At the protein level, reduced expression of CYFIP2 and WAVE1 was observed in whole lysates of the cortex, striatum, and hippocampus but not the cerebellum of adult *Cyfip2* cKO mice (Figures 1C–F), which is consistent with previous studies showing that WAVE protein in the WRC becomes less stable and is degraded by the proteasome without CYFIP (Kunda et al., 2003; Zhao et al., 2013; Han et al., 2015). Notably, CYFIP1 protein expression was increased in the cortex, but not in other brain regions, of *Cyfip2* cKO mice (Figure 1C), suggesting a potential compensatory response of CYFIP1 in the cortex owing to a reduction in CYFIP2.

**Figure 2:**
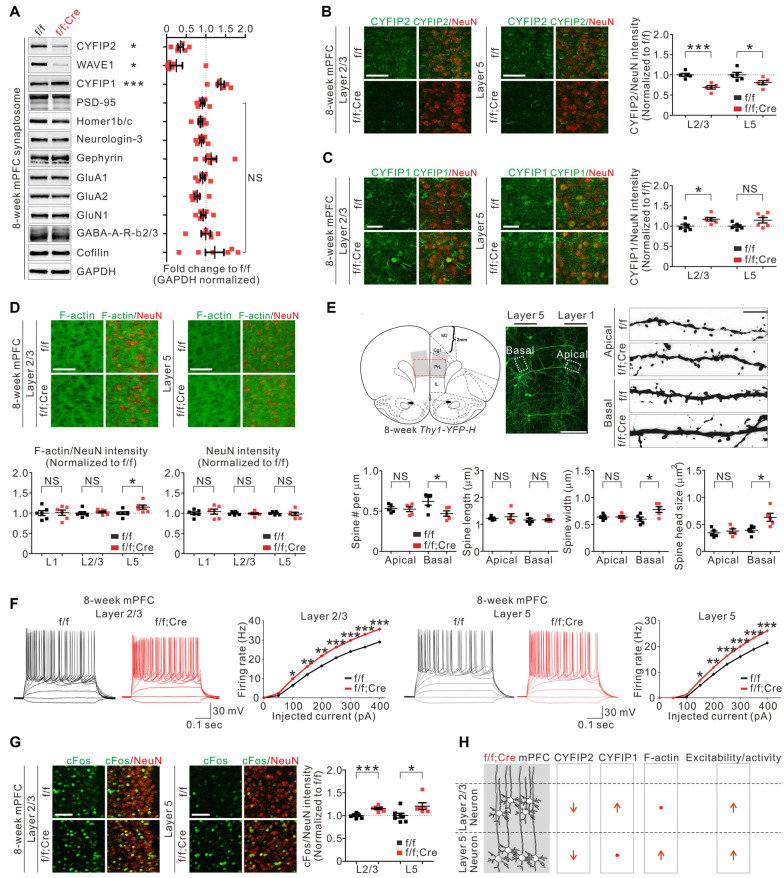
Molecular, morphological, and functional changes in excitatory pyramidal neurons in the medial prefrontal cortex (mPFC) of *Cyfip2* cKO mice. **(A)** Western blot analysis of various synaptic proteins in the mPFC synaptosome of *Cyfip2* cKO mice (*n* = 6 animals per genotype, two-tailed student’s *t*-test). NS, not significant. **(B)** Fluorescence immunohistochemistry (IHC) analysis of CYFIP2 and NeuN in the mPFC of *Cyfip2* cKO mice (*n* = 5 animals per genotype, two-tailed student’s *t*-test). Scale bar, 50 μm. L2/3, layer 2/3; L5, layer 5. **(C)** Fluorescence IHC analysis of CYFIP1 and NeuN in the mPFC of *Cyfip2* cKO mice (*n* = 6 animals per genotype, two-tailed student’s *t*-test). Scale bar, 25 μm. **(D)** Fluorescence IHC analysis of F-actin and NeuN in the mPFC of *Cyfip2* cKO mice (*n* = 6 animals per genotype, two-tailed student’s *t*-test). L1, layer 1. Scale bar, 50 μm. **(E)** Dendritic spine analysis of layer 5 pyramidal neurons in the mPFC of *Cyfip2* cKO mice (*n* = 5 animals per genotype, two-tailed student’s *t*-test). Scale bars, 200 and 5 μm. **(F)** Intrinsic excitabilities of mPFC L2/3 (left panel) and L5 (right panel) neurons measured as firing rates against injected current (*n* = 4 animals per genotype, two-tailed student’s *t*-test). **(G)** Fluorescence IHC analysis of cFos and NeuN in the mPFC of *Cyfip2* cKO mice (*n* = 7 animals for control and 6 for *Cyfip2* cKO, two-tailed student’s *t*-test). Scale bar, 50 μm. **(H)** Summary of molecular and functional changes of pyramidal neurons in the mPFC of *Cyfip2* cKO mice. All data are presented as mean ± SEM. *P < 0.05; **P < 0.01; ***P < 0.001.

To further characterize *Cyfip2* cKO mice, we focused on the mPFC because we recently identified several molecular, cellular, and functional changes in the mPFC excitatory neurons of adult *Cyfip2^+/−^* mice (Lee S. H. et al., 2020). First, we confirmed the decreased CYFIP2 and WAVE1, but increased CYFIP1, protein levels in synaptosomal lysates of adult *Cyfip2* cKO mPFC (Figure 2A). Next, we assessed the levels of several other excitatory and inhibitory synaptic proteins, such as PSD-95 and Gephyrin, and found that they were normal (Figure 2A). We previously showed that in the mPFC of adult *Cyfip2^+/−^* mice, CYFIP2 protein expression is selectively reduced in L5 neurons but not in L2/3 neurons (Lee S. H. et al., 2020). However, in *Cyfip2* cKO mice, we found that CYFIP2 levels were significantly reduced in both L2/3 and L5 neurons of the mPFC (Figure 2B and Supplementary Figure 4). We also observed CYFIP1 expression in both NeuN-positive or CaMKII-positive neurons and NeuN-negative non-neuronal cells in the mPFC of adult wild-type and *Cyfip2* cKO mice (Supplementary Figure 5), which is similar to what we observed in the hippocampus of adult mice (Zhang et al., 2019a). When we selectively analyzed the CYFIP1 signals in NeuN-positive neurons of the mPFC, they were increased in L2/3 neurons, but not in L5 neurons, of *Cyfip2* cKO mice compared with those in control mice (Figure 2C).

In the basal state, CYFIP inhibits WAVE activity of the WRC (Chen et al., 2010) and its downstream actin polymerization (Zhao et al., 2013). As expected, the reduction in CYFIP2 protein observed in adult *Cyfip2^+/−^* mice coincided with an increase of F-actin levels in mPFC L5 neurons (Lee S. H. et al., 2020). Therefore, we also measured F-actin levels in the mPFC of *Cyfip2* cKO mice and found that they were significantly increased in L5 but unchanged in layer 1 (L1) and L2/3 compared with those in control mPFC (Figure 2D). This L5-specific increase of F-actin levels was unexpected because CYFIP2 proteins were reduced in both L2/3 and L5 neurons (Figure 2B). One possible explanation is that the compensatory increase in CYFIP1 (Figure 2C) might normalize the net CYFIP levels in L2/3 neurons of *Cyfip2* cKO mice, thereby maintaining their F-actin levels. NeuN intensities were normal in all layers of *Cyfip2* cKO mPFC (Figure 2D).

Next, we analyzed dendritic spines of the mPFC L5 neurons by crossing *Cyfip2* cKO mice with *Thy1-YFP* mice, which sparsely express yellow fluorescent proteins (YFPs) in L5 neurons visualizing their dendritic spines (Feng et al., 2000). In apical dendrites of L5 neurons, dendritic spine density, and morphology (length, width, and head size) were comparable between control and *Cyfip2* cKO mice (Figure 2E). In contrast, in basal dendrites of L5 neurons, dendritic spine density was decreased, but spine width and head size were increased in *Cyfip2* cKO mice compared with control mice.

We previously showed that, in the mPFC of adult *Cyfip2^+/−^* mice, neuronal excitability was increased in L5, but not in L2/3, neurons (Lee S. H. et al., 2020), suggesting that CYFIP2 negatively regulates neuronal excitability. Consistently, we found that neuronal excitability was increased in both L2/3 and L5 neurons of *Cyfip2* cKO mPFC (Figure 2F). Furthermore, neuronal activity, as measured based on cFos expression levels, was also increased in both L2/3 and L5 neurons of *Cyfip2* cKO mPFC (Figure 2G). It is intriguing that, unlike F-actin levels, which were increased only in L5, neuronal excitability and activity were enhanced in both L2/3 and L5 neurons of *Cyfip2* cKO mPFC. These results suggest that the two functions of CYFIP2 (i.e., regulation of neuronal F-actin and excitability/activity) may be mediated by independent mechanisms and that CYFIP1 can only compensate for the F-actin-related function of CYFIP2 (see our “discussion” below; Figure 2H).

**Figure 3:**
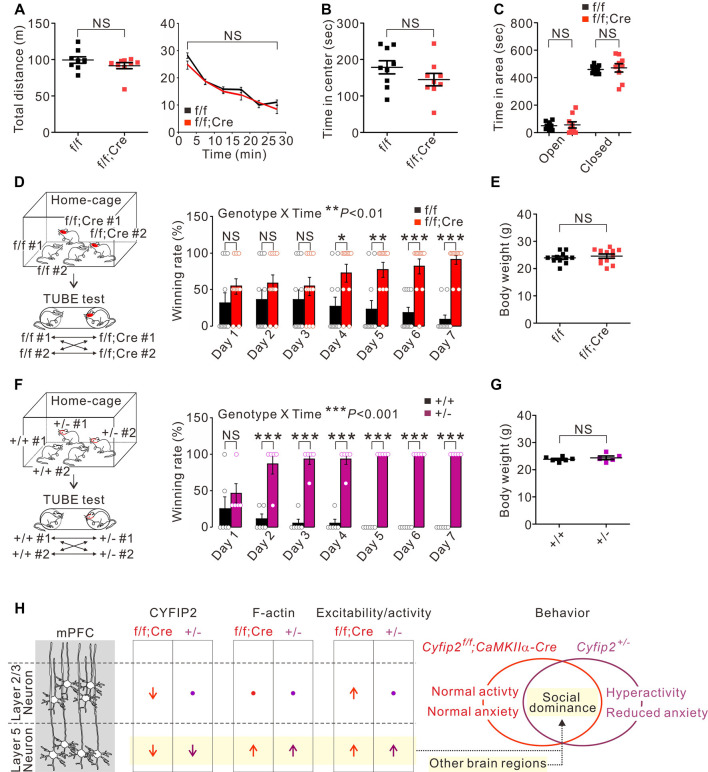
Enhanced social dominance behavior of *Cyfip2* cKO and *Cyfip2^+/−^* mice. **(A)** Locomotor activity of control and *Cyfip2* cKO mice in the open field test (*n* = 9 animals per genotype, two-tailed student’s *t*-test). NS, not significant. **(B)** Time spent in the center area during the open field test for control and *Cyfip2* cKO mice. **(C)** Time spent in the open and closed arms during the elevated plus-maze test for control and *Cyfip2* cKO mice (*n* = 9 animals per genotype, two-tailed student’s *t*-test). **(D)** Social dominance behavior of *Cyfip2* cKO mice measured by the tube test (*n* = 11 animals per genotype, two-way ANOVA with Bonferroni’s *post hoc* test). **(E)** Body weights of control and *Cyfip2* cKO mice were used for the tube test (two-tailed student’s *t*-test). **(F)** Social dominance behavior of *Cyfip2^+/−^* mice measured by the tube test (*n* = 6, 5 wild-type and *Cyfip2^+/−^* mice, respectively, two-way ANOVA with Bonferroni’s *post hoc* test). **(G)** Body weights of wild-type and *Cyfip2^+/−^* mice used for the tube test (two-tailed student’s *t*-test). **(H)** Summary of molecular and functional changes of pyramidal neurons in the mPFC of *Cyfip2* cKO and *Cyfip2^+/−^* mice, and a hypothesis linking the changes in layer 5 neurons to increased social dominance behavior of the mice. Changes in other brain regions may also contribute to the phenotype. All data are presented as mean ± SEM. *P < 0.05; **P < 0.01; ***P < 0.001.

Next, we characterized behavioral phenotypes of adult *Cyfip2* cKO mice. Based on the locomotor hyperactivity and reduced anxiety observed in adult *Cyfip2^+/−^* mice (Han et al., 2015; Lee S. H. et al., 2020), we performed the open field test and elevated plus maze test in adult *Cyfip2* cKO mice. However, unlike *Cyfip2^+/−^* mice, *Cyfip2* cKO mice displayed normal locomotor activity (Figure 3A), and anxiety (Figures 3B,C) levels. Changes in synaptic strength and neuronal activity in the mPFC are causally associated with social dominance behavior in mice (Wang et al., 2011, 2014). Therefore, we performed the tube test (Fan et al., 2019) to measure the social hierarchy among the group-housed *Cyfip2* cKO mice and their control littermates (Figure 3D). Notably, we found that throughout the 7-day tube test, *Cyfip2* cKO mice displayed a significantly higher winning rate over control mice from day 4 to day 7. There was no significant difference in body weights between the two groups (Figure 3E). These results prompted us to test the social dominance behavior in adult *Cyfip2^+/−^* mice (Figure 3F). Like *Cyfip2* cKO mice, *Cyfip2^+/−^* mice displayed a significantly higher winning rate over their control littermates from day 2 to day 7 of the tube test. Body weights were similar between the two genotypes (Figure 3G). Altogether, these results suggest enhanced social dominance behavior in both adult *Cyfip2^+/−^* mice and *Cyfip2* cKO mice.

In this study, we generated *Cyfip2* cKO mice with reduced CYFIP2 expression selectively in postnatal forebrain excitatory neurons. Specifically, in the mPFC, CYFIP2 protein levels were significantly reduced in L2/3 and L5 neurons of adult *Cyfip2* cKO mice. Intriguingly, however, F-actin levels were increased only in L5, but not L2/3, neurons of *Cyfip2* cKO mPFC, possibly because of a compensatory increase in CYFIP1 proteins in L2/3 neurons. This increase in CYFIP1 may substitute for CYFIP2 in the WRC of L2/3 neurons, thereby maintaining the basal levels of WRC activity and its downstream actin polymerization. However, unlike the L5-specific increase in F-actin levels, neuronal excitability and activity were enhanced in both L2/3 and L5 neurons of *Cyfip2* cKO mPFC. Therefore, it is conceivable that the observed increase in CYFIP1 in L2/3 neurons is not sufficient to compensate for increased neuronal excitability and activity induced by a reduction in CYFIP2. This also suggests that the function of CYFIP2 regulating neuronal excitability and activity may be independent of the WRC activity and F-actin levels at least in L2/3 neurons.

Beyond the WRC, CYFIP1 and CYFIP2 can interact with a diverse range of proteins in the brain, such as RNA-binding proteins (RBPs) and postsynaptic scaffolding proteins (De Rubeis et al., 2013; Kumar et al., 2013; Li et al., 2017; Lee Y. et al., 2020). Notably, we recently showed that CYFIP1 and CYFIP2 can interact with distinct types of RBPs (Lee Y. et al., 2020). Specifically, among the 25 RBPs identified from the neonatal mouse forebrain CYFIP2 interactome, only two were found in common with the CYFIP1 brain interactome. Whether these differential interactions with RBPs contribute to the CYFIP2-specific roles in regulating neuronal excitability and activity is unclear. Future studies aimed at identifying target mRNAs of the CYFIP1-RBP and CYFIP2-RBP complexes and characterizing their functional implications in neurons are needed.

To better understand molecular mechanisms possibly contributing to the differential phenotypes of L2/3 and L5 neurons of *Cyfip2* cKO mPFC, we investigated the relative mRNA expression levels between the forebrain L2/3 and L5 neurons for the 140 proteins of mouse forebrain CYFIP2 interactome (Lee Y. et al., 2020). Specifically, we used a recently established mouse brain single-cell gene expression database (Saunders et al., 2018) where we could get the relative mRNA expression ratios (L2/3 to L5) for 118 out of 140 proteins of the CYFIP2 interactome (Supplementary Table 2). We found that 39 out of 118 CYFIP2 interactors showed relatively higher mRNA expression in L2/3 neurons than in L5 neurons (L2/3-high), while 31 out of 118 CYFIP2 interactors showed the opposite relative expression ratio (L5-high). GO and pathway analyses on the L2/3-high 39 proteins revealed a significant enrichment of actin-related terms (Supplementary Table 3). Indeed, all key components of the WRC were included in the L2/3-high 39 proteins. However, no significant term was identified from the same analyses on the L5-high 31 proteins (Supplementary Table 4). These results suggest that the mPFC L2/3 neurons express relatively higher levels of mRNAs of actin-related CYFIP2 interactors than L5 neurons, and thus may be more resilient to CYFIP2 reduction in terms of maintaining normal F-actin levels (e.g., a compensatory increase in CYFIP1 proteins (Figure 2C) which were possibly translated from the abundant *Cyfip1* mRNAs). Further experiments are necessary to validate this intriguing, yet premature, hypothesis.

Synaptic strength and neuronal activity in the mPFC are causally associated with social dominance status in mice (Wang et al., 2014). Specifically, an increase and decrease in excitatory synaptic efficacy in the mPFC cause an upward and downward change in social rank, respectively (Wang et al., 2011). Furthermore, direct optogenetic activation of the mPFC induces instantaneous winning in the tube test, while inhibition causes the opposite effect (Zhou et al., 2017; Tan et al., 2018). It has been proposed that, at the circuit level, the activation or inhibition of the mPFC modulates its downstream target regions including the striatum, basolateral amygdala, and dorsal raphe nucleus, thereby inducing changes in the social dominance behavior (Wang et al., 2014). Among the mPFC neurons, the deepest layer 5/6 neurons are the main output neurons targeting such subcortical nuclei (Riga et al., 2014). Considering the above-mentioned findings, it is conceivable that enhanced neuronal excitability/activity and/or increased F-actin levels of the mPFC L5 neurons may contribute to this enhanced social dominance behavior in adult *Cyfip2^+/−^* mice and *Cyfip2* cKO mice (Figure 3H). The effect of F-actin increases on social dominance cannot be ignored because the involvement of the mPFC actin dynamics in social dominance behavior in mice has recently been reported (Tada et al., 2016). Importantly, neuronal changes in other brain regions, such as other PFC and subcortical areas, should also be carefully investigated to clearly understand critical brain regions and neuronal types involved in social dominance behavior of *Cyfip2^+/−^* mice and *Cyfip2* cKO mice (Figure 3H).

It is somewhat counterintuitive that *Cyfip2* cKO mice with lower CYFIP2 expressions in both L2/3 and L5 neurons did not display locomotor hyperactivity or reduced anxiety as observed in *Cyfip2^+/−^* mice considering that they also have lower CYFIP2 expressions but only in L5 neurons. One possible explanation is that the reduction in CYFIP2 in other neuronal cell types, such as inhibitory neurons, may contribute to such behavioral phenotypes in *Cyfip2^+/−^* mice. Indeed, we previously showed that β-galactosidase signals expressed from the LacZ cassette of *Cyfip2^+/−^* mice, which represent the promoter activity of the *Cyfip2* gene, were detected in parvalbumin-positive inhibitory neurons of the mPFC (Lee S. H. et al., 2020). Therefore, CYFIP2 expression is also affected in inhibitory neurons of *Cyfip2^+/−^* mice, but not in *Cyfip2* cKO mice that express Cre recombinase only in CaMKIIα-positive excitatory neurons. Another possibility is that this occurs because of the early developmental effects of CYFIP2 reduction in *Cyfip2^+/−^* mice. That is, brain CYFIP2 expression decreases in the early embryonic stages in *Cyfip2^+/−^* mice, whereas this decrease does not occur until the third to fourth postnatal week following the onset of Cre expression in *Cyfip2* cKO mice. We previously showed that mRNA and protein levels of *Cyfip2* were lowered by approximately 50% in the cortex of embryonic day 18.5 *Cyfip2^+/−^* mice (Zhang et al., 2018). Such a long-term reduction in CYFIP2 in *Cyfip2^+/−^* L5 neurons may have a larger effect on the associated brain circuits, thereby leading to more diverse behavioral phenotypes than those observed in *Cyfip2* cKO mice. It is a prerequisite for validating the above-mentioned hypothesis to carefully characterize the spatiotemporal and cell-type-specific expression changes of CYFIP2 and its interactors, such as WAVE1, in both *Cyfip2^+/−^* mice and *Cyfip2* cKO mice. Also, more comprehensive investigations on the cell-type- and age-specific brain functions of CYFIP2 are necessary, with a special emphasis on those factors implicated in the early-onset characteristics of *CYFIP2*-associated brain disorders (Zhong et al., 2019). To that end, our floxed-*Cyfip2* mice represent a useful tool that can be combined with various available Cre-lines (Tsien, 2016) to more thoroughly assess other cell types of different developmental stages.

In conclusion, our results provide additional evidence that supports the critical roles CYFIP2 plays in regulating actin dynamics and neuronal excitability/activity in the mPFC principal neurons. Further investigations on the various mPFC target regions in *Cyfip2* cKO mice and *Cyfip2^+/−^* mice may provide better insights into understanding the circuit-level mechanisms involved in the social dominance behavior of mice.

## Data Availability Statement

The original contributions presented in the study are included in the article/Supplementary Material, further inquiries can be directed to the corresponding authors.

## Ethics Statement

The animal study was reviewed and approved and all procedures were approved by the Committee on Animal Research at Korea University College of Medicine (KOREA-2018-0174).

## Author Contributions

YZ, HRK, S-HL, YKi, RM, CJ, J-EL, SK, YKa, SYK, S-KK, S-YC, and KH designed and performed the experiments. HRK, S-YC, and KH analyzed and interpreted the data. KH wrote the article. All authors contributed to the article and approved the submitted version.

## Conflict of Interest

The authors declare that the research was conducted in the absence of any commercial or financial relationships that could be construed as a potential conflict of interest.
