# Supplementary material for: Enhanced Prefrontal Neuronal Activity and Social Dominance Behavior in Postnatal Forebrain Excitatory Neuron-Specific Cyfip2 Knock-Out Mice
Source: Front Mol Neurosci. 2020 Oct 29;13:574947. doi: 10.3389/fnmol.2020.574947 (PMC7658541; doi:10.3389/fnmol.2020.574947)
Supplement: Supplementary file 1 [file Table_1.DOCX]

**Supplementary material**

**Enhanced prefrontal neuronal activity and social dominance behavior in postnatal forebrain excitatory neuron-specific *Cyfip2* knock-out mice**

Yinhua Zhang, Hyae Rim Kang, Seung-Hyun Lee, Yoonhee Kim, Ruiying Ma, Chunmei Jin, Ji-Eun Lim, Seoyeon Kim, Yeju Kang, Hyojin Kang, Su Yeon Kim, Seok-Kyu Kwon, Se-Young Choi, and Kihoon Han

**List of Supplementary Figures and Tables**

Figure S1. The mouse brain coordinates of immunohistochemical analyses performed in this study.

Figure S2. Examination of antibody penetration for immunohistochemical analyses of the mPFC sections.

Figure S3. Description of the procedures for image quantification of immunohistochemical analyses.

Figure S4. Fluorescence immunohistochemical analysis of CYFIP2 and CaMKII in the mPFC of control and *Cyfip2* cKO mice.

Figure S5. CYFIP1 expression in both NeuN-positive or CaMKII-positive neurons and NeuN-negative non-neuronal cells in the mPFC.

Table S1. List of primary and secondary antibodies used for immunohistochemistry.

Table S2. Relative mRNA expression levels of proteins in the CYFIP2 interactome between mouse forebrain L2/3 and L5 neurons.

Table S3. Gene ontology and pathway analyses for the L2/3-high 39 proteins of CYFIP2 interactome.

Table S4. Gene ontology and pathway analyses for the L5-high 31 proteins of CYFIP2 interactome.

**
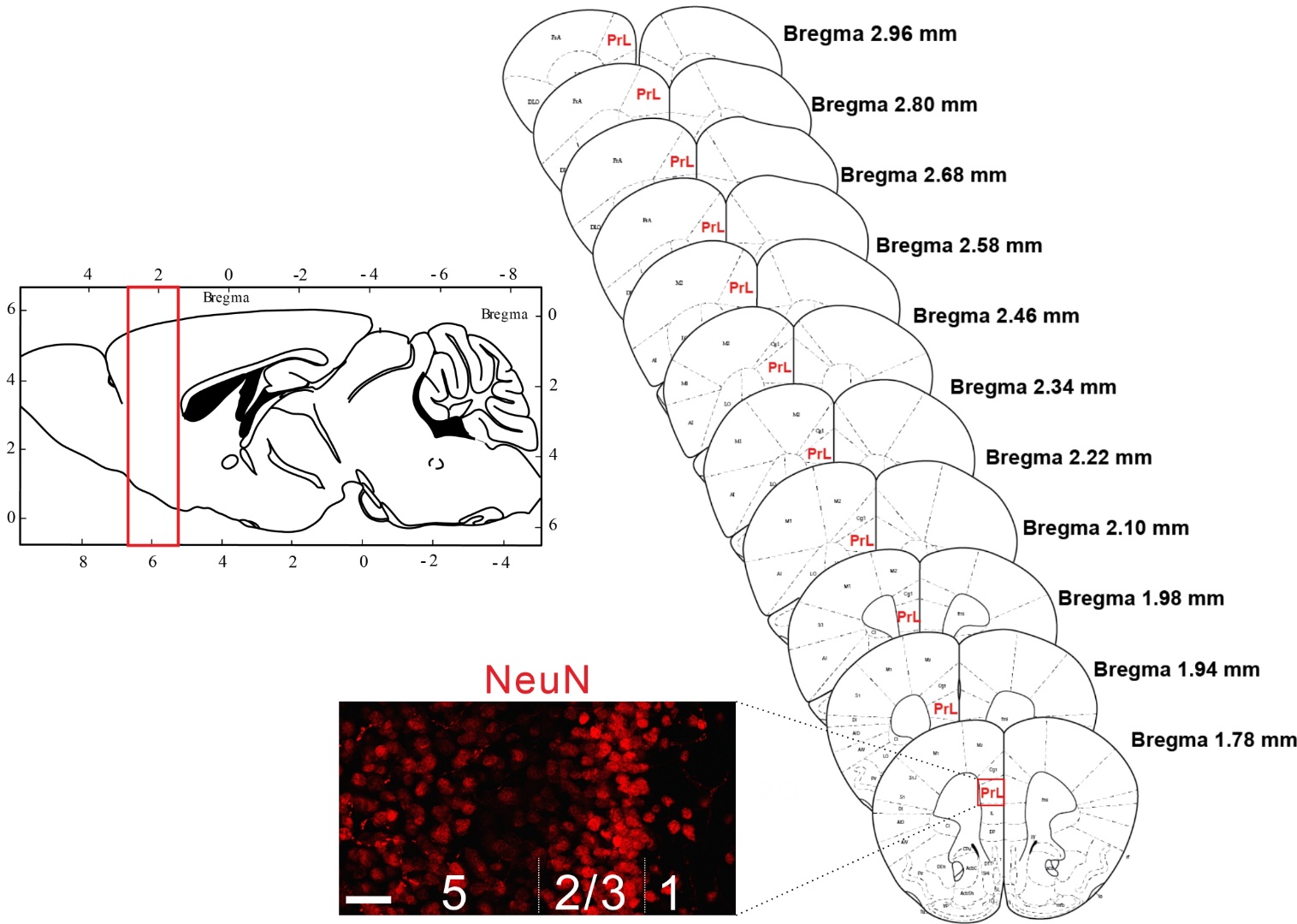
**

**Figure S1. The mouse brain coordinates of immunohistochemical analyses performed in this study.** As described in the methods section of the main article, the prelimbic (PrL) region of the medial prefrontal cortex (mPFC) (3.00-1.77 mm anterior from the bregma) was imaged from coronal sections. Sample image of NeuN fluorescence immunohistochemistry is shown. Numbers indicate layers. Scale bar, 50 μm.

**
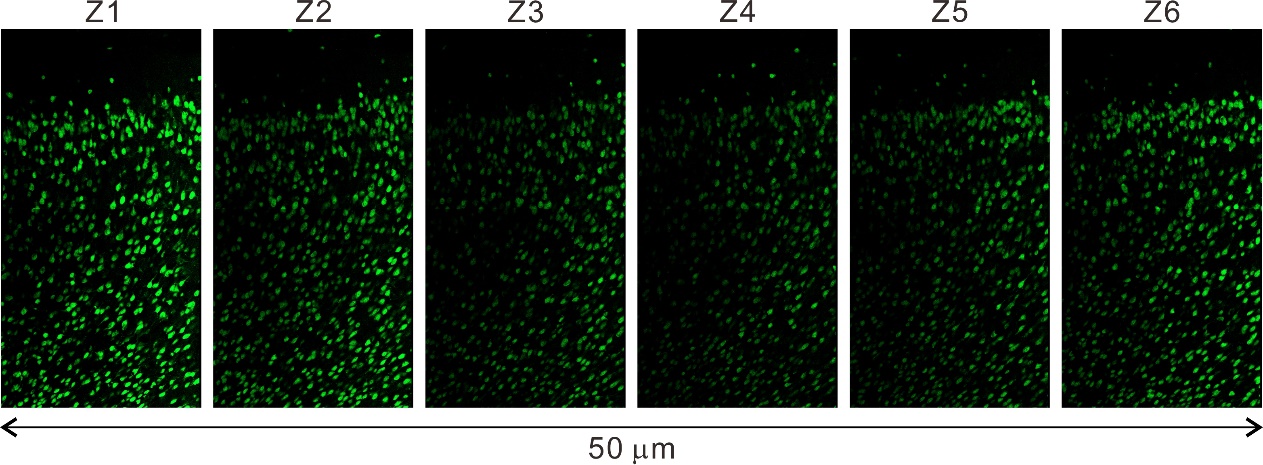
**

**Figure S2. Examination of antibody penetration for immunohistochemical analyses of the mPFC sections.** NeuN fluorescence immunohistochemical signals from each frame of Z-stacks (total six stacks across 50 μm) are shown.

**
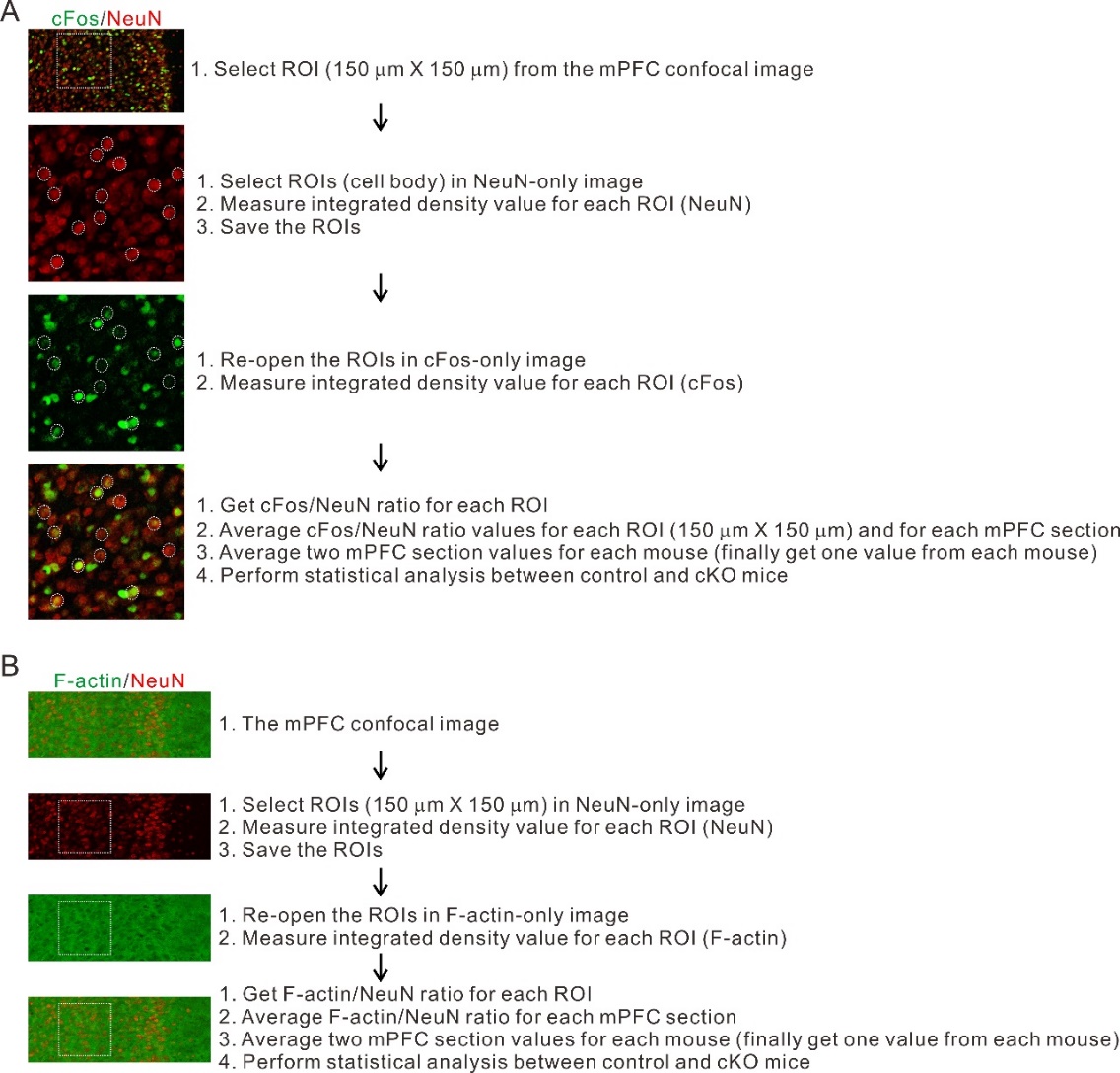
**

**Figure S3. Description of the procedures for image quantification of immunohistochemical analyses. (A)** Quantification of cFos levels in the mPFC. The same procedures were applied to quantification of CYFIP1 levels to selectively measure CYFIP1 signals in NeuN-positive neurons, not NeuN-negative non-neuronal cells (**Supplementary Figure S5**). **(B)** Quantification of F-actin levels in the mPFC. The same procedures were applied to quantification of CYFIP2 levels.

**
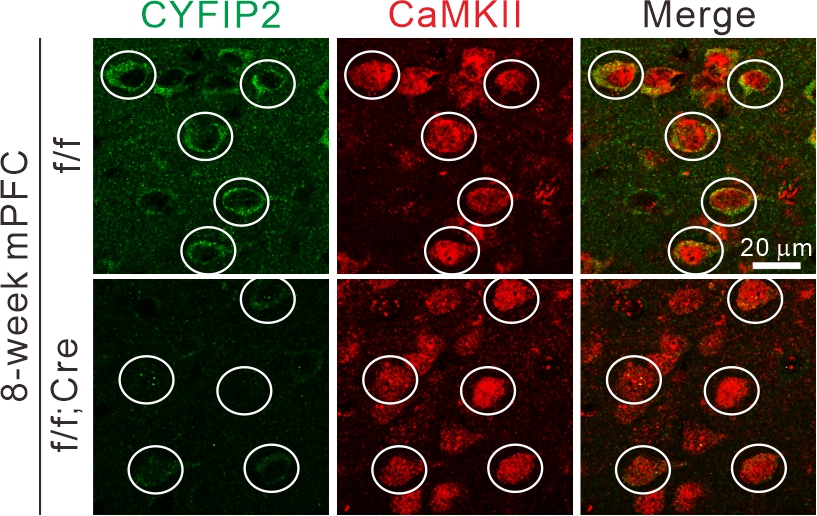
**

**Figure S4.** **Fluorescence immunohistochemical analysis of CYFIP2 and CaMKII in the mPFC of control and *Cyfip2* cKO mice.** CYFIP2 signals were decreased in CaMKII-positive excitatory neurons (circles) of *Cyfip2* cKO mice (f/f;Cre) compared with those of control mice (f/f).

**
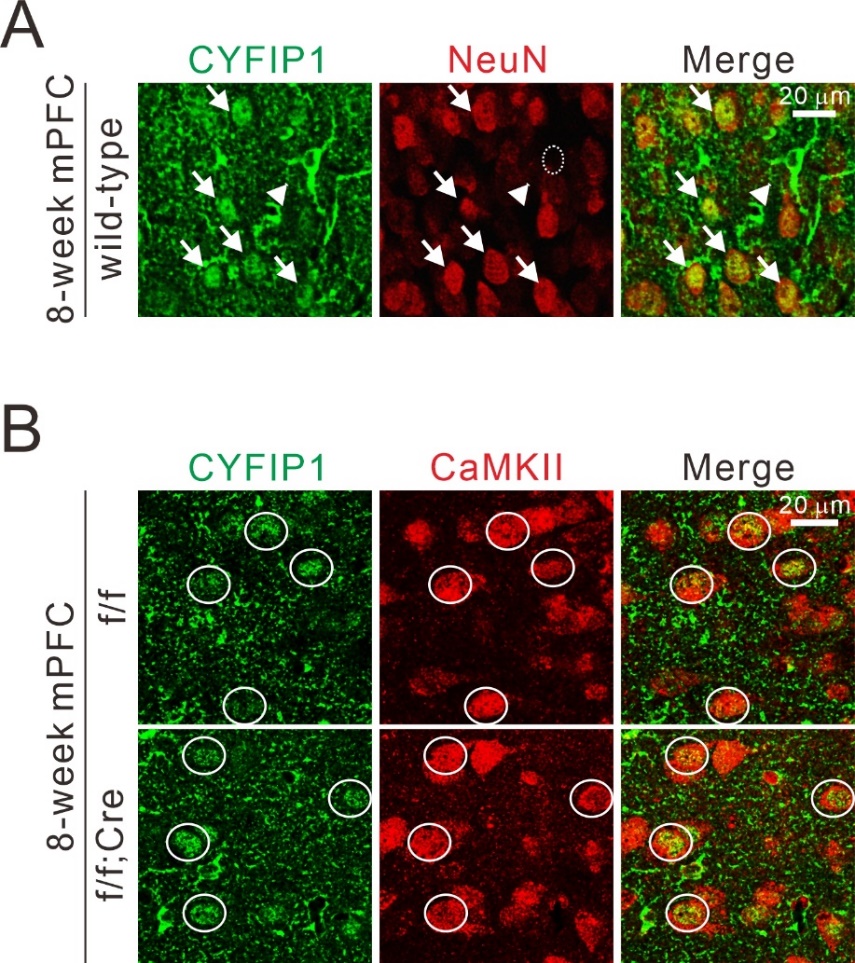
**

**Figure S5. CYFIP1 expression in both NeuN-positive or CaMKII-positive neurons and NeuN-negative non-neuronal cells in the mPFC. (A)** Fluorescent immunohistochemistry using anti-CYFIP1 and anti-NeuN antibodies on the mPFC of adult wild-type mice shows CYFIP1 signals in NeuN-positive neurons (arrows) as well as in NeuN-negative non-neuronal cells (arrowhead). **(B)** Fluorescent immunohistochemistry using anti-CYFIP1 and anti-CaMKII antibodies in the mPFC of control (f/f) and *Cyfip2* cKO (f/f;Cre) mice shows expression of CYFIP1 in CaMKII-positive neurons (circles) in both mice.

**Table S1. List of primary and secondary antibodies used for immunohistochemistry.** All secondary antibodies were purchased from Jackson Immunoresearch Laboratories, and diluted in a ratio of 1:500.

| **Figure #** | **Primary antibody** | **Secondary antibody** |
| --- | --- | --- |
| Figure 2B | Anti-CYFIP2 (Rabbit, abcam, ab95969 1: 200)  Anti-NeuN (Mouse, Millipore, MAB377, 1:1000) | Anti-rabbit Alexa Fluor-488  Anti-mouse Alexa Fluor-594 |
| Figure 2C | Anti-CYFIP1 (Rabbit, Millipore, AB6046, 1:500)  Anti-NeuN (Mouse, Millipore, MAB377, 1:1000) | Anti-rabbit Alexa Fluor-488  Anti-mouse Alexa Fluor-594 |
| Figure 2D | Alexa Fluor 488 Phalloidin (Invitrogen, A-12379, 1:40)  Anti-NeuN (Mouse, Millipore, MAB377, 1:1000) | Anti-mouse Alexa Fluor-594 |
| Figure 2E | Anti-GFP (Chicken, abcam, ab13970, 1:1000) | Anti-chicken Alexa Fluor-488 |
| Figure 2G | Anti-cFos (Rabbit, Synaptic System, 226-003, 1:500)  Anti-NeuN (Mouse, Millipore, MAB377, 1:1000) | Anti-rabbit Alexa Fluor-488  Anti-mouse Alexa Fluor-594 |
| Figure S4 | Anti-CYFIP2 (Rabbit, abcam, ab95969 1: 200)  Anti-CaMKII (Mouse, Thermo, MA 1-048, 1:100) | Anti-rabbit Alexa Fluor-488  Anti-mouse Alexa Fluor-594 |
| Figure S5B | Anti-CYFIP1 (Rabbit, Millipore, AB6046, 1:500)  Anti-CaMKII (Mouse, Thermo, MA 1-048, 1:100) | Anti-rabbit Alexa Fluor-488  Anti-mouse Alexa Fluor-594 |

**Table S2. Relative mRNA expression levels of proteins in the CYFIP2 interactome between mouse forebrain L2/3 and L5 neurons.** Expression values were obtained from the DropViz (<http://dropviz.org/>) database. The L2/3-high 39 proteins are highlighted in red, and the L5-high 31 proteins are highlighted in blue. Components of the WAVE regulatory complex are in bold.

| **Region** | **Frontal Cortex** | **Frontal Cortex** |  |
| --- | --- | --- | --- |
| **Class** | **Neuron** | **Neuron** |  |
| **Cluster** | **Neuron_Layer2/3_Nptxr [#6]** | **Neuron_Layer5_Parm1 [#7]** | **Ratio** |
| Nes Amount | 0.693147181 | 0 |  |
| Atp1b2 Amount | 1.945910149 | 0.693147181 | 2.807355 |
| Bphl Amount | 1.098612289 | 0.693147181 | 1.584963 |
| Dnpep Amount | 1.098612289 | 0.693147181 | 1.584963 |
| Tbcd Amount | 1.098612289 | 0.693147181 | 1.584963 |
| Clpx Amount | 1.386294361 | 1.098612289 | 1.26186 |
| Gtf2f2 Amount | 1.386294361 | 1.098612289 | 1.26186 |
| Usp12 Amount | 1.386294361 | 1.098612289 | 1.26186 |
| C2cd2l Amount | 2.708050201 | 2.197224577 | 1.232487 |
| Ndufs4 Amount | 1.945910149 | 1.609437912 | 1.209062 |
| Amph Amount | 2.48490665 | 2.079441542 | 1.194988 |
| Ubxn6 Amount | 2.079441542 | 1.791759469 | 1.160558 |
| Rps6ka3 Amount | 2.197224577 | 1.945910149 | 1.12915 |
| Dnajc7 Amount | 2.890371758 | 2.564949357 | 1.126873 |
| Cobl Amount | 3.044522438 | 2.708050201 | 1.124249 |
| Ppp2cb Amount | 2.564949357 | 2.302585093 | 1.113943 |
| Sema7a Amount | 1.791759469 | 1.609437912 | 1.113283 |
| Cops7a Amount | 2.302585093 | 2.079441542 | 1.107309 |
| Sun1 Amount | 2.302585093 | 2.079441542 | 1.107309 |
| Ttyh3 Amount | 2.302585093 | 2.079441542 | 1.107309 |
| Strn4 Amount | 2.63905733 | 2.397895273 | 1.100572 |
| **Abi1 Amount** | **2.397895273** | **2.197224577** | **1.091329** |
| **Cyfip1 Amount** | **1.945910149** | **1.791759469** | **1.086033** |
| Ptpn5 Amount | 2.833213344 | 2.63905733 | 1.07357 |
| Dgkz Amount | 3.610917913 | 3.36729583 | 1.072349 |
| Gng3 Amount | 3.401197382 | 3.17805383 | 1.070214 |
| **Wasf1 Amount** | **3.258096538** | **3.044522438** | **1.07015** |
| Fam117b Amount | 2.079441542 | 1.945910149 | 1.068622 |
| **Nckap1 Amount** | **3.761200116** | **3.526360525** | **1.066595** |
| Usp5 Amount | 2.63905733 | 2.48490665 | 1.062035 |
| **Brk1 Amount** | **2.995732274** | **2.833213344** | **1.057362** |
| Eif5 Amount | 3.688879454 | 3.496507561 | 1.055018 |
| Zc3h15 Amount | 3.891820298 | 3.688879454 | 1.055014 |
| Sec62 Amount | 4.343805422 | 4.204692619 | 1.033085 |
| Pum1 Amount | 3.496507561 | 3.401197382 | 1.028023 |
| **Abi2 Amount** | **3.688879454** | **3.610917913** | **1.021591** |
| Celf1 Amount | 4.276666119 | 4.189654742 | 1.020768 |
| Nipbl Amount | 3.091042453 | 3.044522438 | 1.01528 |
| Taok3 Amount | 3.17805383 | 3.135494216 | 1.013573 |
| **Cyfip2 Amount** | **4.369447852** | **4.356708827** | **1.002924** |
| Ablim3 Amount | 0.693147181 | 0.693147181 | 1 |
| Ago1 Amount | 1.609437912 | 1.609437912 | 1 |
| Ago2 Amount | 2.197224577 | 2.197224577 | 1 |
| Aimp2 Amount | 1.098612289 | 1.098612289 | 1 |
| Arhgap33 Amount | 1.791759469 | 1.791759469 | 1 |
| Atl2 Amount | 1.609437912 | 1.609437912 | 1 |
| Cbx4 Amount | 1.609437912 | 1.609437912 | 1 |
| Cttn Amount | 1.609437912 | 1.609437912 | 1 |
| Dcakd Amount | 1.098612289 | 1.098612289 | 1 |
| Dnajc11 Amount | 1.609437912 | 1.609437912 | 1 |
| Dpm3 Amount | 1.098612289 | 1.098612289 | 1 |
| Fbll1 Amount | 1.609437912 | 1.609437912 | 1 |
| Gja1 Amount | 0.693147181 | 0.693147181 | 1 |
| Hmgn1 Amount | 1.609437912 | 1.609437912 | 1 |
| Kazn Amount | 1.098612289 | 1.098612289 | 1 |
| Kpna4 Amount | 1.945910149 | 1.945910149 | 1 |
| Letm1 Amount | 2.397895273 | 2.397895273 | 1 |
| Lmbrd2 Amount | 2.197224577 | 2.197224577 | 1 |
| Map1s Amount | 1.609437912 | 1.609437912 | 1 |
| Mbd3 Amount | 1.945910149 | 1.945910149 | 1 |
| Mpc2 Amount | 2.48490665 | 2.48490665 | 1 |
| Mrpl28 Amount | 1.791759469 | 1.791759469 | 1 |
| Mta3 Amount | 0.693147181 | 0.693147181 | 1 |
| Myo16 Amount | 0.693147181 | 0.693147181 | 1 |
| Ocrl Amount | 1.791759469 | 1.791759469 | 1 |
| Pabpn1 Amount | 1.609437912 | 1.609437912 | 1 |
| Pip5k1a Amount | 2.708050201 | 2.708050201 | 1 |
| Plgrkt Amount | 0.693147181 | 0.693147181 | 1 |
| Pnkp Amount | 0.693147181 | 0.693147181 | 1 |
| Ppp1r12c Amount | 2.079441542 | 2.079441542 | 1 |
| Prpf4 Amount | 1.098612289 | 1.098612289 | 1 |
| Prr12 Amount | 1.609437912 | 1.609437912 | 1 |
| Rbbp6 Amount | 3.465735903 | 3.465735903 | 1 |
| Rundc3b Amount | 1.386294361 | 1.386294361 | 1 |
| Sar1a Amount | 2.48490665 | 2.48490665 | 1 |
| Senp3 Amount | 1.386294361 | 1.386294361 | 1 |
| Sf3a1 Amount | 1.791759469 | 1.791759469 | 1 |
| Sgpl1 Amount | 0.693147181 | 0.693147181 | 1 |
| Slc27a1 Amount | 0.693147181 | 0.693147181 | 1 |
| Spast Amount | 1.791759469 | 1.791759469 | 1 |
| Specc1l Amount | 1.386294361 | 1.386294361 | 1 |
| Srp68 Amount | 1.609437912 | 1.609437912 | 1 |
| Tdrd3 Amount | 0.693147181 | 0.693147181 | 1 |
| Tmem87a Amount | 1.098612289 | 1.098612289 | 1 |
| Ublcp1 Amount | 1.609437912 | 1.609437912 | 1 |
| **Wasf2 Amount** | **0.693147181** | **0.693147181** | **1** |
| Xrn2 Amount | 2.48490665 | 2.48490665 | 1 |
| Tcf25 Amount | 4.672828834 | 4.691347882 | 0.996053 |
| Prnp Amount | 4.574710979 | 4.605170186 | 0.993386 |
| Myo9a Amount | 3.610917913 | 3.63758616 | 0.992669 |
| Mia3 Amount | 3.091042453 | 3.135494216 | 0.985823 |
| Dlgap4 Amount | 3.36729583 | 3.433987204 | 0.980579 |
| Ntm Amount | 4.820281566 | 4.934473933 | 0.976858 |
| Dag1 Amount | 2.708050201 | 2.772588722 | 0.976723 |
| Ccdc47 Amount | 2.63905733 | 2.708050201 | 0.974523 |
| Bzw2 Amount | 2.397895273 | 2.48490665 | 0.964984 |
| Far1 Amount | 2.397895273 | 2.48490665 | 0.964984 |
| Calr Amount | 4.025351691 | 4.17438727 | 0.964298 |
| Mfn2 Amount | 2.302585093 | 2.397895273 | 0.960253 |
| Plcl2 Amount | 2.302585093 | 2.397895273 | 0.960253 |
| Acox1 Amount | 2.197224577 | 2.302585093 | 0.954243 |
| Elavl3 Amount | 4.356708827 | 4.574710979 | 0.952346 |
| Chd5 Amount | 4.060443011 | 4.343805422 | 0.934766 |
| Matk Amount | 3.044522438 | 3.295836866 | 0.923748 |
| Ncln Amount | 1.791759469 | 1.945910149 | 0.920782 |
| Gria3 Amount | 4.532599493 | 4.927253685 | 0.919904 |
| Tomm40 Amount | 2.48490665 | 2.708050201 | 0.9176 |
| Tnr Amount | 2.944438979 | 3.295836866 | 0.893381 |
| Ralgapb Amount | 1.945910149 | 2.197224577 | 0.885622 |
| Sacm1l Amount | 1.791759469 | 2.079441542 | 0.861654 |
| Scai Amount | 2.302585093 | 2.708050201 | 0.850274 |
| Phf6 Amount | 1.098612289 | 1.386294361 | 0.792481 |
| Vipas39 Amount | 1.098612289 | 1.386294361 | 0.792481 |
| Arhgef1 Amount | 0.693147181 | 1.098612289 | 0.63093 |
| Kctd8 Amount | 0.693147181 | 1.098612289 | 0.63093 |
| Nup93 Amount | 0.693147181 | 1.098612289 | 0.63093 |
| Sorbs1 Amount | 0.693147181 | 1.098612289 | 0.63093 |
| Vash1 Amount | 0 | 0.693147181 | 0 |

**Table S3. Gene ontology and pathway analyses for the L2/3-high 39 proteins of CYFIP2 interactome.** Significant terms are shown in bold.

| **Category** | **Term** | **Overlap** | **P-value** | **Adjusted P-value** | **Odds Ratio** | **Combined Score** | **Genes** |
| --- | --- | --- | --- | --- | --- | --- | --- |
| Gene Ontology  Biological  Process | **Regulation of Arp2/3 complex-mediated actin nucleation (GO:0034315)** | **5/10** | **5.40E-12** | **2.76E-08** | **256.41** | **6652.3** | **CYFIP1;NCKAP1;ABI2;BRK1;WASF1** |
|  | **Positive regulation of actin nucleation (GO:0051127)** | **5/13** | **2.75E-11** | **7.01E-08** | **197.239** | **4796.36** | **CYFIP1;NCKAP1;ABI2;BRK1;WASF1** |
|  | **Rac protein signal transduction (GO:0016601)** | **5/19** | **2.46E-10** | **4.19E-07** | **134.953** | **2985.82** | **CYFIP1;NCKAP1;ABI2;BRK1;WASF1** |
|  | **Actin polymerization or depolymerization (GO:0008154)** | **4/36** | **6.95E-07** | **8.87E-04** | **56.9801** | **807.938** | **ABI2;ABI1;COBL;WASF1** |
|  | **Vascular endothelial growth factor receptor signaling pathway (GO:0048010)** | **4/70** | **1.03E-05** | **0.01053** | **29.304** | **336.463** | **CYFIP1;NCKAP1;ABI1;BRK1** |
| Gene Ontology  Molecular Function | **Rac GTPase binding (GO:0048365)** | **5/43** | **1.97E-08** | **2.27E-05** | **59.6303** | **1058** | **CYFIP1;NCKAP1;ABI2;BRK1;WASF1** |
|  | **Rho GTPase binding (GO:0017048)** | **5/72** | **2.75E-07** | **1.58E-04** | **35.6125** | **538.004** | **CYFIP1;NCKAP1;ABI2;BRK1;WASF1** |
|  | ATPase activator activity (GO:0001671) | 2/23 | 9.13E-04 | 0.350454 | 44.59309 | 312.0758 | DNAJC7;ATP1B2 |
|  | ATPase regulator activity (GO:0060590) | 2/38 | 0.002492 | 0.716995 | 26.99055 | 161.8024 | DNAJC7;ATP1B2 |
|  | mRNA binding (GO:0003729) | 3/179 | 0.005085 | 1 | 8.594757 | 45.39238 | CELF1;PUM1;ZC3H15 |
| Gene Ontology  Cellular Component | Filopodium tip (GO:0032433) | 2/10 | 1.65E-04 | 0.07363 | 102.5641 | 893.2329 | ABI2;ABI1 |
|  | Filopodium (GO:0030175) | 2/60 | 0.006107 | 1 | 17.09402 | 87.15213 | ABI2;ABI1 |
|  | Polymeric cytoskeletal fiber (GO:0099513) | 3/221 | 0.009077 | 1 | 6.961364 | 32.73233 | TBCD;COBL;NES |
|  | Cytoskeleton (GO:0005856) | 4/520 | 0.018108 | 1 | 3.944773 | 15.82411 | AMPH;WASF1;GTF2F2;NES |
|  | Actin cytoskeleton (GO:0015629) | 3/294 | 0.019456 | 1 | 5.232862 | 20.61533 | COBL;AMPH;WASF1 |
| Kyoto Encyclopedia of Genes and Genomes  (KEGG) | **Regulation of actin cytoskeleton** | **5/214** | **5.73E-05** | **0.01765** | **11.9818** | **117.028** | **CYFIP1;NCKAP1;ABI2;BRK1;WASF1** |
|  | Fc gamma R-mediated phagocytosis | 2/91 | 0.013602 | 1 | 11.27078 | 48.43659 | AMPH;WASF1 |
|  | Choline metabolism in cancer | 2/99 | 0.015957 | 1 | 10.36001 | 42.8685 | WASF1;DGKZ |
|  | MAPK signaling pathway | 3/295 | 0.01963 | 1 | 5.215124 | 20.49907 | RPS6KA3;TAOK3;PTPN5 |
|  | Oocyte meiosis | 2/125 | 0.024698 | 1 | 8.205128 | 30.3675 | RPS6KA3;PPP2CB |

**Table S4. Gene ontology and pathway analyses for the L5-high 31 proteins of CYFIP2 interactome.**

| **Category** | **Term** | **Overlap** | **P-value** | **Adjusted P-value** | **Odds Ratio** | **Combined Score** | **Genes** |
| --- | --- | --- | --- | --- | --- | --- | --- |
| Gene Ontology  Biological  Process | Negative regulation of cell migration (GO:0030336) | 4/121 | 3.53E-05 | 0.180356 | 21.32765 | 218.617 | DAG1;MIA3;CALR;SCAI |
|  | Negative regulation of cell motility (GO:2000146) | 3/97 | 4.50E-04 | 1 | 19.95344 | 153.7475 | DAG1;MIA3;SCAI |
|  | Protein destabilization (GO:0031648) | 2/31 | 0.001051 | 1 | 41.62331 | 285.442 | PRNP;NCLN |
|  | Negative regulation of Ras protein signal transduction (GO:0046580) | 2/37 | 0.001497 | 1 | 34.87358 | 226.8274 | MFN2;SCAI |
|  | Establishment of protein localization to mitochondrion (GO:0072655) | 2/49 | 0.002613 | 1 | 26.33311 | 156.6098 | TOMM40;MFN2 |
| Gene Ontology  Molecular Function | Amyloid-beta binding (GO:0001540) | 2/49 | 0.002613 | 1 | 26.33311 | 156.6098 | PRNP;GRIA3 |
|  | Protein transporter activity (GO:0008565) | 2/80 | 0.006815 | 1 | 16.12903 | 80.46168 | TOMM40;MIA3 |
|  | Tubulin binding (GO:0015631) | 3/255 | 0.007074 | 1 | 7.590133 | 37.58118 | PRNP;DAG1;PHF6 |
|  | Phosphatidylinositol phosphate phosphatase activity (GO:0052866) | 1/6 | 0.009265 | 1 | 107.5269 | 503.3873 | SACM1L |
|  | Cuprous ion binding (GO:1903136) | 1/6 | 0.009265 | 1 | 107.5269 | 503.3873 | PRNP |
| Gene Ontology  Cellular Component | Intrinsic component of mitochondrial outer membrane (GO:0031306) | 2/22 | 5.27E-04 | 0.234955 | 58.65103 | 442.7378 | TOMM40;MFN2 |
|  | Peroxisomal matrix (GO:0005782) | 2/42 | 0.001926 | 0.429506 | 30.72197 | 192.0827 | ACOX1;FAR1 |
|  | Microbody lumen (GO:0031907) | 2/42 | 0.001926 | 0.286337 | 30.72197 | 192.0827 | ACOX1;FAR1 |
|  | Peroxisomal part (GO:0044439) | 2/75 | 0.006014 | 0.67051 | 17.2043 | 87.97834 | ACOX1;FAR1 |
|  | Endoplasmic reticulum lumen (GO:0005788) | 3/270 | 0.008273 | 0.737914 | 7.168459 | 34.37139 | DAG1;MIA3;CALR |
| Kyoto Encyclopedia of Genes and Genomes  (KEGG) | PPAR signaling pathway | 2/74 | 0.005859 | 1 | 17.43679 | 89.62177 | ACOX1;SORBS1 |
|  | ECM-receptor interaction | 2/82 | 0.007149 | 1 | 15.73564 | 77.74747 | DAG1;TNR |
|  | Peroxisome | 2/83 | 0.007318 | 0.751313 | 15.54606 | 76.4465 | ACOX1;FAR1 |
|  | Alpha-Linolenic acid metabolism | 1/25 | 0.03806 | 1 | 25.80645 | 84.35078 | ACOX1 |
|  | Biosynthesis of unsaturated fatty acids | 1/27 | 0.041043 | 1 | 23.89486 | 76.29926 | ACOX1 |
